# Quality of vital event data for infant mortality estimation in prospective, population-based studies: an analysis of secondary data from Asia, Africa, and Latin America

**DOI:** 10.1186/s12963-023-00309-7

**Published:** 2023-07-28

**Authors:** Daniel J. Erchick, Seema Subedi, Andrea Verhulst, Michel Guillot, Linda S. Adair, Aluísio J. D. Barros, Bernard Chasekwa, Parul Christian, Bruna Gonçalves C. da Silva, Mariângela F. Silveira, Pedro C. Hallal, Jean H. Humphrey, Lieven Huybregts, Simon Kariuki, Subarna K. Khatry, Carl Lachat, Alicia Matijasevich, Peter D. McElroy, Ana Maria B. Menezes, Luke C. Mullany, Tita Lorna L. Perez, Penelope A. Phillips-Howard, Dominique Roberfroid, Iná S. Santos, Feiko O. ter Kuile, Thulasiraj D. Ravilla, James M. Tielsch, Lee S. F. Wu, Joanne Katz

**Affiliations:** 1 Department of International Health, Johns Hopkins Bloomberg School of Public Health, 615 N. Wolfe Street, Baltimore, MD 21205 USA; 2 Population Studies Center, University of Pennsylvania, Philadelphia, PA USA; 3 Department of Sociology, University of Pennsylvania, Philadelphia, PA USA; 4 Carolina Population Center, University of North Carolina at Chapel Hill, Chapel Hill, NC USA; 5 Postgraduate Program in Epidemiology, Federal University of Pelotas, Pelotas, Brazil; 6 Zvitambo Institute for Maternal and Child Health Research, Harare, Zimbabwe; 7 Poverty, Health and Nutrition Division, International Food Policy Research Institute, Washington, DC USA; 8 Kenya Medical Research Institute/Centre for Global Health Research, Kisumu, Kenya; 9 Nepal Nutrition Intervention Project – Sarlahi, Kathmandu, Nepal; 10 Department of Food Technology, Safety and Health, Faculty of Bioscience Engineering, Ghent University, Ghent, Belgium; 11 Department of Preventive Medicine, Faculty of Medicine FMUSP, University of São Paulo, São Paulo, Brazil; 12 Malaria Branch, Centers for Disease Control and Prevention, Atlanta, GA USA; 13 USC-Office of Population Studies Foundation, University of San Carlos, Cebu City, Philippines; 14 Faculty of Medicine, Namur University, Namur, Belgium; 15 Department of Clinical Sciences, Liverpool School of Tropical Medicine, Pembroke Place, Liverpool, UK; 16 Aravind Eye Care System, Madurai, India; 17 Department of Global Health, Milken Institute School of Public Health, George Washington University, Washington, DC USA

## Abstract

**Introduction:**

Infant and neonatal mortality estimates are typically derived from retrospective birth histories collected through surveys in countries with unreliable civil registration and vital statistics systems. Yet such data are subject to biases, including under-reporting of deaths and age misreporting, which impact mortality estimates. Prospective population-based cohort studies are an underutilized data source for mortality estimation that may offer strengths that avoid biases.

**Methods:**

We conducted a secondary analysis of data from the Child Health Epidemiology Reference Group, including 11 population-based pregnancy or birth cohort studies, to evaluate the appropriateness of vital event data for mortality estimation. Analyses were descriptive, summarizing study designs, populations, protocols, and internal checks to assess their impact on data quality. We calculated infant and neonatal morality rates and compared patterns with Demographic and Health Survey (DHS) data.

**Results:**

Studies yielded 71,760 pregnant women and 85,095 live births. Specific field protocols, especially pregnancy enrollment, limited exclusion criteria, and frequent follow-up visits after delivery, led to higher birth outcome ascertainment and fewer missing deaths. Most studies had low follow-up loss in pregnancy and the first month with little evidence of date heaping. Among studies in Asia and Latin America, neonatal mortality rates (NMR) were similar to DHS, while several studies in Sub-Saharan Africa had lower NMRs than DHS. Infant mortality varied by study and region between sources.

**Conclusions:**

Prospective, population-based cohort studies following rigorous protocols can yield high-quality vital event data to improve characterization of detailed mortality patterns of infants in low- and middle-income countries, especially in the early neonatal period where mortality risk is highest and changes rapidly.

**Supplementary Information:**

The online version contains supplementary material available at 10.1186/s12963-023-00309-7.

## Introduction

Infant and neonatal mortality rates are important indicators of trends in child health that serve to inform global health policy and programs [1]. Complete civil registration and vital statistics systems (CRVS systems) that collect continuous data on birth and death events are common sources of mortality data as they provide comprehensive, high-quality, and timely estimates of mortality. Yet there are gaps in our knowledge of mortality among infants in many low- and middle-income countries (LMICs) due to incomplete or inaccurate CRVS systems [2]. Only two-thirds (68%) of countries globally have CRVS systems that record data on at least 90% of all deaths; this threshold is met by only 25% of countries in South Asia and 8% in Sub-Saharan Africa, compared to 83% in Latin America and the Caribbean [3]. Further, detailed, high-quality data on patterns of mortality by age are not widely available beyond the traditional cut-offs of infant (birth to 1 year) and neonatal (birth to 28 days) periods, especially in the first week of life [4, 5].

In countries without strong CRVS systems, mortality estimates are typically derived from retrospective full birth histories (FBH) collected through sample surveys such as the United States Agency for International Development (USAID)-supported Demographic and Health Surveys (DHS) and UNICEF-supported Multiple Indicator Cluster Surveys (MICS) [6, 7]. However, although commonly used for many settings without reliable CRVS systems [8, 9], birth histories are subject to biases, including under-reporting of deaths and age misreporting that can impact mortality estimates, with the strongest effects occurring early in the neonatal period [10, 11]. New approaches are needed to estimate how infant and neonatal mortality are distributed by detailed age strata in settings where mortality is high and CRVS systems are inadequate.

Many maternal and child health population-based studies prospectively enroll and follow a pregnancy or birth cohort that could be used for mortality estimation. Although these studies, which are typically randomized controlled trials (RCTs) and observational prospective cohort studies, aim to evaluate the effect of a specific intervention or measure associations between suspected risk factors and child health outcomes, they have strengths that could avoid shortcomings and biases associated with DHS data [12]. Cohort studies often include prospective, systematic follow-up, protocols that might reduce biases common in the DHS’s retrospective FBHs, such as non-response bias, recall biases, and date heaping. Health and Demographic Surveillance Systems (HDSS), which collect longitudinal data though regular surveys in a defined geographic area and population, are another important source of health data in countries without strong CRVS systems. Compared to HDSSs, which utilize prospective annual or semi-annual visits, cohort studies often have frequent household visits to collect detailed information on vital events during critical periods, such as during pregnancy and the perinatal period.

Cohort studies also have potential weaknesses and data quality issues. Cohorts are conducted in limited geographic areas, similar to HDSS and different from the DHS, which may not necessarily represent the national population. Some cohort studies span a short period, in contrast to HDSS sites, which operate continuously, exposing cohorts to biases associated with seasonality and unusual external events (e.g., famine). Despite the shortcomings of HDSS and DHS, their continuity (although not always in the same season) offers the benefit of evaluating public health trends over time compared to cohorts, which are high cost and transient. Communities where cohort studies are based often selected because they have higher mortality, thereby reducing sample size requirements and allowing for an understanding of how interventions function in settings where they are most needed. Further, these studies are designed to look at specific research questions and; therefore, sometimes utilize inclusion/exclusion criteria that may not lead to the enrollment of a representative sample in the geographic study area. Study visit protocols can determine whether a very high proportion of deaths are identified, including whether and when pregnancies or births are enrolled, facility delivery rates in the study area, how quickly the study team makes home visits after the birth, and how live births and stillbirths are classified. Understanding these factors is required to determine the accuracy of mortality estimates obtained from a specific cohort.

Unlike DHS or HDSS, cohort studies are not a commonly used data source for mortality estimates in LMICs. The goal of this study was to evaluate the potential of this underutilized source of information for the purpose of mortality estimation and understanding detailed patterns of mortality by age. We assessed the effect of common data quality issues on infant and neonatal mortality measurement in several population-based pregnancy or birth cohort studies from Asia, Sub-Saharan Africa, and Latin America. We suggest approaches to prevent, measure, and control for these issues in understanding patterns of mortality in these populations.

## Methods

We conducted a secondary analysis of data from the Child Health Epidemiology Reference Group (CHERG), including population-based cohort studies from Asia, Sub-Saharan Africa, and Latin America [13]. The goal of CHERG was to generate evidence to support estimation of child mortality burden and causes of death. We selected 11 CHERG studies that included individual-level participant vital event data for this descriptive analysis. We evaluated the appropriateness of vital event data for mortality estimation and understanding detailed mortality patterns in three dimensions. The first dimension comprises the study design, population, and field protocols, which is subdivided into study design and population (i.e., randomized controlled trial or cohort study; enrollment of pregnancies or live births), inclusion/exclusion criteria, surveillance and enrollment protocols, and visit frequency. The second and third dimensions included, internal data quality checks (loss to follow-up (LTF) (both in pregnancy and the infant period), date heaping, and shape of the mortality curve) and external checks (comparison with DHS), respectively.

Of 11 studies in our analysis, seven enrolled women in pregnancy, and four enrolled live births. For each of the seven studies that enrolled pregnancies, we summarized the number of pregnancies identified, the number of pregnancies enrolled, and the number of pregnancies followed to a birth outcome. We defined an unknown birth outcome as a confirmed pregnancy (i.e., typically a positive urine test, excluding false positive results) for which no information on the outcome of the birth was available to the study investigators. Reasons for LTF in pregnancy were classified as the following: 1) withdrawal of consent, 2) out-migration, censoring, or could not be contacted, 3) maternal death during pregnancy with unknown birth outcome, or 4) data entry or management error leading to loss of information on the birth outcome. Birth outcomes, including live births, stillbirths, and miscarriages/abortions, were defined as classified by the original study investigators.

For all 11 studies, we summarized the number of infant deaths, number of surviving infants, and number of infants LTF for both the neonatal (0–< 28 days) and infant periods (0–< 365 days). LTF in the neonatal and infant periods was defined as an infant for which the study investigators did not know the vital status of the infant at the end of the time period.

For each of the 11 studies, we identified DHS data for comparison by selecting the DHS survey with the closest time period relative to the study follow-up and the DHS region with the closest geographical location relative to the study site (see the footnote to Table 3 for the specific DHS surveys and regions that we selected). Within each DHS survey dataset, we restricted the analysis population to birth outcomes that occurred in the selected region within the years matching the data collection period for the respective cohort study. For example, if a cohort study began enrollment sometime in 2004 and followed the last participant until sometime in 2009, we included DHS participants with births occurring between January 1, 2004, and December 31, 2009. For each participant, we assigned an exit date as the date death or date of interview if this event occurred before December 31, 2009, or we administratively censored participant follow-up at December 31, 2009, if the event occurred after this date.

Cohort studies and DHS allowed us to use the same method for computing mortality estimates. In both cases, we used individual data to compute age-specific death rates by week for the neonatal period (0–6 days; 7–13 days; 14–20 days; 21–27 days) and by month for the post-neonatal period (months 2–12) using the event/exposure approach presented by Hill (2013) [14]. We calculated age-specific death rates by dividing the number of deaths by the person-years computed in each age group and for the corresponding period. We then cumulated these deaths rates—under the assumption of a constant force of mortality within each age interval—to obtained cumulative probabilities of dying from birth to age 28 days and one year, namely the so-called neonatal (NMR) and the infant (IMR) mortality rates. We presented these cumulative probabilities of dying as number of deaths per 1000 live births.

We graphed the distribution of LTF over time for the neonatal and infant periods against distribution of infants who died and log age-specific mortality rates, respectively, to visually assess the potential extent and timing of missed mortality outcomes due to LTF. We displayed the frequency of each day of the month for dates of birth and death in histograms to visually explore evidence of date heaping.

Datasets shared with our research team by the original study investigators contained no identifying information and; therefore, this analysis was considered exempt by the Institutional Review Board at the Johns Hopkins Bloomberg School of Public Health.

## Results

### Study design, population, and field protocols

#### Study design and population

We utilized 11 studies in this analysis, including seven RCTs and four observational longitudinal cohort studies, from Asia (*n *= 4), Sub-Saharan Africa (*n *= 4), and Latin America (*n *= 3) conducted between 1983 and 2015. Seven studies enrolled and followed pregnant women and their infants, and four enrolled only live births, yielding a total of 71,760 pregnant women and 85,095 live births for analysis. Studies were population-based, either recruiting pregnancies or live births through census and systematic surveillance of households in a community or regular surveillance of health facilities in a geographical area with a rate of facility of delivery > 90% (Table 1).

**Table 1:** Study characteristics

Study	Setting	Study design	Study population	Primary outcome	Inclusion/exclusion criteria	Systematic follow-up period	Methods for pregnancy surveillance	Study follow-up frequency in pregnancy	Methods for birth outcome reporting or identifying live births	Study follow-up frequency after birth outcome	Cohort type
*Asia*											
India 2000	Rural Tamil Nadu	RCT of newborn vitamin A supplementation	Recruitment of all pregnant women in study area	6-month mortality	All women identified as pregnant in the study area were included	6 months	Local data collectors identified pregnancies from a variety of sources, including community-based health workers, antenatal care clinics, and other development workers in the study area	Visited every 2 weeks in pregnancy and at least once or twice a week in the last month of pregnancy	Local data collectors reported births. Mothers and infants were visited on the day of birth or as soon as possible afterward	Birth visit < 48 h; every two weeks until 6 months	Open pregnancy cohort
Nepal 1999	Rural Sarlahi	Cluster RCT of multiple micronutrient supplementation	Recruitment of all pregnant women in study area	Fetal loss and infant mortality	Exclusion criteria: women currently pregnant, breastfeeding an infant aged 9 month, were sterilized, were menopausal, or whose husband had died	> 1 year	Household census to identify eligible women. Local data collectors conducted visits at home every 5 weeks for pregnancy surveillance	Only one baseline visit after positive pregnancy test	Local data collectors reported births. Mothers and infants were visited on the day of birth or as soon as possible afterward	Birth visit < 72 h; daily visits in first 10 days; weekly visits in first 3 months; 6-, 12- months; 8-year visit	Open pregnancy cohort
Nepal 2011	Rural Sarlahi	RCT of newborn mustard oil massage	Recruitment of all pregnant women in study area	Neonatal mortality	Inclusion criteria: married women 15–40 years of age	28 days	Household census to identify eligible women. Local data collectors conducted visits at home every 5 weeks for pregnancy surveillance	Monthly visits in pregnancy after positive pregnancy test and visit at 32-weeks	Local data collectors reported births. Mothers and infants were visited on the day of birth or as soon as possible afterward	Birth visit < 72 h; visits on 1-, 3-, 7-, 10-, 14-, 21-, and 28- days	Open pregnancy cohort
Philippines 1983	Urban Cebu	Longitudinal health-nutritional study of infant feeding patterns	Population-based, random cluster sample of census of pregnant women	Infant feeding patterns, determinants, and outcomes	Inclusion criteria: women who lived in the study area and delivered a single live birth	> 1 year	Household census to identify eligible women	Only one baseline survey in 6th month of pregnancy	Midwives and traditional birth attendants working in the survey area were hired to report all births. Reports made were verified by the study staff	Birth visit on 3rd day after delivery (conducted even for infant deaths < 3 days); every two months from months 2 to 24	Open pregnancy cohort
*Sub-Saharan Africa*											
Burkina Faso 2004	Rural Hounde	RCT of multiple micronutrient supplementation	Prospective, community-based cohort of pregnant women	Fetal growth outcomes	Exclusion criteria: plan to leave the area within the next 2 years	1 year	Household census to identify eligible women. Local data collectors conducted visits at home every month for pregnancy surveillance	Daily visits in pregnancy	Local data collectors reported births	Birth visit < 24 h; monthly postnatal visits at clinic	Closed pregnancy cohort
Burkina Faso 2006	Rural Hounde	RCT of micronutrient fortified balanced energy–protein supplementation	Prospective, community-based cohort of pregnant women	Fetal growth outcomes	Exclusion criteria: plan to leave the area within the next 2 years	1 year	Household census to identify eligible women. Local data collectors conducted visits at home every month for pregnancy surveillance	Daily visits in pregnancy	Local data collectors reported births	Birth visit < 24 h; monthly postnatal visits at clinic	Closed pregnancy cohort
Kenya 1992*	Rural Western Kenya	RCT of insecticide treated nets	Prospective, community-based cohort of pregnant women	Under-five mortality	All resident pregnant women and their newborns were eligible for enrollment	> 1 year	Monthly censuses by trained village monitors and/or trained traditional birth attendants residing in the same village	Monthly visits in pregnancy; weekly visits in final month of pregnancy	Traditional birth attendants monitored birth outcomes and visited the household within 24 h after delivery	First visit < 24 h after delivery; end of week 1 and week 2, every 2 weeks until either 2 or 5 years of age	Open pregnancy cohort
Zimbabwe 1997	Urban Harare	RCT of maternal-neonatal vitamin A supplementation	Facility-based recruitment of live births from 14 maternity clinics and hospital	Infant mortality	Eligible participants: no acutely life-threatening condition in mother or infant; singleton infant with birth weight ≥ 1500 g; mother planned to stay in Harare after delivery	1 year	N/A	N/A	Women were recruited in the clinic following delivery of a live born infant	First visit < 96 h after delivery; 6 weeks; 3-, 6-, 9-, 12- months. Some also visited at 15-, 18-, 21-, and 24-months	Closed live birth cohort
*Latin America*											
Brazil 1993	Urban Pelotas	Longitudinal birth cohort study	Facility-based recruitment of all births in Pelotas hospitals	Multiple maternal and child health indicators	Inclusion criteria: living in the urban area of Pelotas	> 1 year	N/A	N/A	All maternity hospitals in the city were visited daily to identify live births	Visits at birth; 1-, 3-, 6-, 12-, 48- months; 11-, 15-, 18-, 22- years	Closed live birth cohort
Brazil 2004	Urban Pelotas	Longitudinal birth cohort study	Facility-based recruitment of all births in Pelotas hospitals	Multiple maternal and child health indicators	Inclusion criteria: living in the urban area of Pelotas	> 1 year	N/A	N/A	All maternity hospitals in the city were visited daily	Visits at birth; 3-, 12-, 24-, and 48-months; 6-, 11-, 15-, 18- years	Closed live birth cohort
Brazil 2015	Urban Pelotas	Longitudinal pregnancy cohort study	Facility-based recruitment of pregnancies and all births in Pelotas hospitals	Multiple maternal and child health indicators	Inclusion criteria: living in the urban area of Pelotas	> 1 year	Pregnancies were identified through weekly visits to, or other contact with, 123 health facilities conducted by study staff	Study visit conducted between 16 and 24 weeks gestation	Research teams were stationed at the four largest hospitals where > 99% of births in the city occur; daily visits to the fifth hospital, where the remaining births take place, were conducted by a mobile team	Visits at birth within 1–2 days after delivery; 3-, 12-, 24-, and 48-months	Open pregnancy cohort

*In Kenya 1992, data collected in two cohorts were pooled for this analysis; these included an observational cohort between 1992 and 1996 and trial of insecticide treated nets between 1997 and 1999

#### Inclusion/exclusion criteria

Most studies had broad inclusion criteria and few exclusion criteria, which were related primarily to residency in the study area and posed minimal potential to bias vital event data (India 2000 [15], Nepal 1999 16, Nepal 2011 [17], Burkina Faso 2004 [18] and 2006 [19], Kenya 1992 [20, 21], and Brazil 1993 [22], 2004 [23], 2015 [24]). Zimbabwe 1997 [25], however, excluded mothers or infants with acutely life-threatening conditions, infants with birth weight < 1500 g, and multiple births. The Philippines 1983 [26] excluded multiple births.

#### Surveillance and enrollment protocols

Most community-based studies conducted a single census survey to either identify and follow women of reproductive age or immediately enroll currently pregnant women (Nepal 1999, Nepal 2011, Philippines 1983, Burkina Faso 2004 and 2006). India 2000 identified pregnancies from various sources, including community-based health workers, antenatal care clinics, and development workers in the study area. Kenya 1992 utilized monthly censuses by trained village monitors and/or traditional birth attendants to identify and enroll pregnancies. Brazil 2015, a facility-based study, identified pregnancies through weekly contact with 123 health facilities conducted by study staff. For studies enrolling pregnancies, outcomes were typically reported by locally-resident study staff (Nepal 1999, India 2000, Nepal 2011, Burkina Faso 2004 and 2006). The Philippines 1983 and Kenya 1992 utilized non-study traditional birth attendants to report birth outcomes and study staff to conduct enrollment, birth, and other follow-up visits. Brazil 1993 and 2004 and Zimbabwe 1997 enrolled only live births (i.e., not pregnant women) through visits by study staff to health facilities in the study area (notably, Zimbabwe 1997 only enrolled women during the day, not at night).

#### Visit frequency

Follow-up visits in pregnancy to identify birth outcomes ranged from very frequent (daily visits in Burkina Faso 2004 and 2006) to infrequent (one baseline visit in pregnancy before delivery in the Philippines 1983). First visits for ascertainment of the birth outcome ranged from < 96 h (Zimbabwe 1997) to the day of birth (Brazil 1992, 2004, and 2015), although most (*n *= 10) studies conducted this visit < 72 h and half of the studies (*n *= 6) at < 24 h after delivery. The frequency of follow-up visits in the early days and weeks of life ranged from daily visits in the first ten days of life (Nepal 1999) to a visit at three months after the initial birth visit (Brazil 2004 and 2015).

### Internal data quality checks

#### Lost to follow-up during pregnancy

Studies enrolled a high proportion of the pregnancies identified through surveillance (97.6% to 100%) (Table 2). After pregnancy enrollment, LTF before the birth outcome was low for most studies (0% to 13.0%). Reasons for LTF in pregnancy included refusal/withdrawal of consent (0% to 2.5%); out-migration, participant unreachable, or participant missed by birth outcome surveillance (< 0.1% to 13.1%); maternal death (0% to 0.2%); and data error issues from (0% to 0.5%) (Additional file 1: Appendix 1).

**Table 2:** Identifying and recording pregnancies, loss to follow-up in pregnancy, and birth outcomes

Study	No. pregnancies identified	No. (%) pregnancies enrolled*	No. (%) pregnancies followed to birth outcome +	No. (%) pregnancies with unknown birth outcome +	No. of birth outcomes	No. (%) live births ~	No. (%) stillbirths ~	No. (%) miscarriages/abortions ~
*Asia*								
India 2000	14,026	14,026 (100.0%)	13,255 (94.5%)	771 (5.5%)	13,376	12,936 (96.7%)	358 (2.7%)	82 (0.6%)
Nepal 1999	4992	4992 (100.0%)	4985 (99.9%)	7 (0.1%)	5019	4130 (82.3%)	156 (3.1%)	733 (14.6%)
Nepal 2011	42,472	42,050 (99.0%)	36,595 (86.2%)	5455 (12.8%)^1^	36,874	32,121 (87.1%)	903 (2.4%)	1795 (4.9%)^2^
Philippines 1983	3711	3702 (99.8%)	3220 (86.8%)	482 (13.0%)^3^	3257	3149 (96.7%)	40 (1.2%)	68 (2.1%)
*Sub-Saharan Africa*^*‡*^								
Burkina Faso 2004	1426	1424 (99.9%)	1381 (96.8%)	43 (3.0%)	1406	1337 (95.1%)	35 (2.5%)	34 (2.4%)
Burkina Faso 2006	1297	1296 (99.9%)	1270 (97.9%)	26 (2.0%)	1293	1225 (94.7%)	27 (2.1%)	41 (3.2%)
*Latin America*								
Brazil 2015	4374^4^	4270 (97.6%)	4270 (97.6%)	0 (0.0%)	4329	4275 (98.8%)	54 (1.2%)	0 (0.0%)

^*^Pregnancies enrolled as a proportion of those identified expressed as a percent

+ Pregnancies followed or not followed to the birth outcome, respectively, as a proportion of pregnancies enrolled expressed as a percent

~ Specific birth outcomes as a proportion of total birth outcomes expressed as a percent. Birth outcomes differ from the number of pregnancies followed to the birth outcome because of the occurrence of multiple births (i.e., twins or triplets)

^‡^Kenya 1992 enrolled and followed pregnancies, however, we only had access to live births for this secondary analysis

^1^In Nepal 2011, 3101 (55.8%) of pregnancies lost to follow-up were censored due to the end of the study

^2^Included in the number of birth outcomes for Nepal 2011 in this table are 2055 outcomes, which were identified as either miscarriage or stillbirth, but could not be stratified further

^3^A total of 3327 pregnant women completed a baseline enrollment visit and were followed by the Cebu Longitudinal Health and Nutrition Survey Study. Of these pregnant women, 3179 (95.6%) completed a birth outcome ascertainment visit. Data on the remainder of the 3711 eligible pregnant women and their infants were collected through later follow-up surveys

^4^Not included in the number of pregnancies for Brazil 2015 are 1227 incomplete pregnancies, stillbirths, or pregnancies not eligible at time of delivery (i.e., residing and/or delivering outside the cohort catchment area, not completing the pregnancy, delivering before 1 January 2015 or after 31 December 2015). Only pregnancy outcomes occurring in 2015 among the women in the pregnancy cohort were eligible for the perinatal study, which provided the data for our analysis

#### Loss to follow-up in infant period

**Fig. 1:**
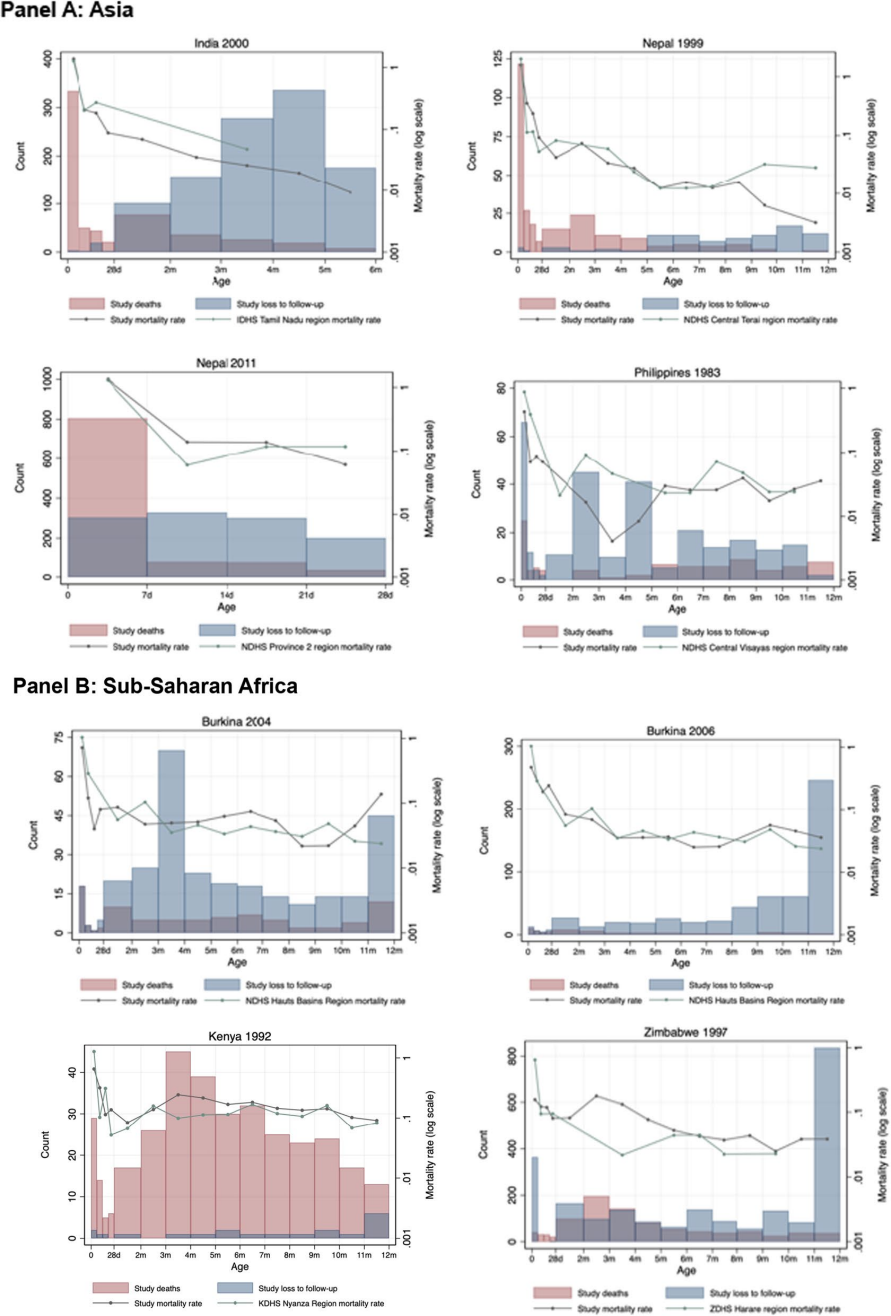

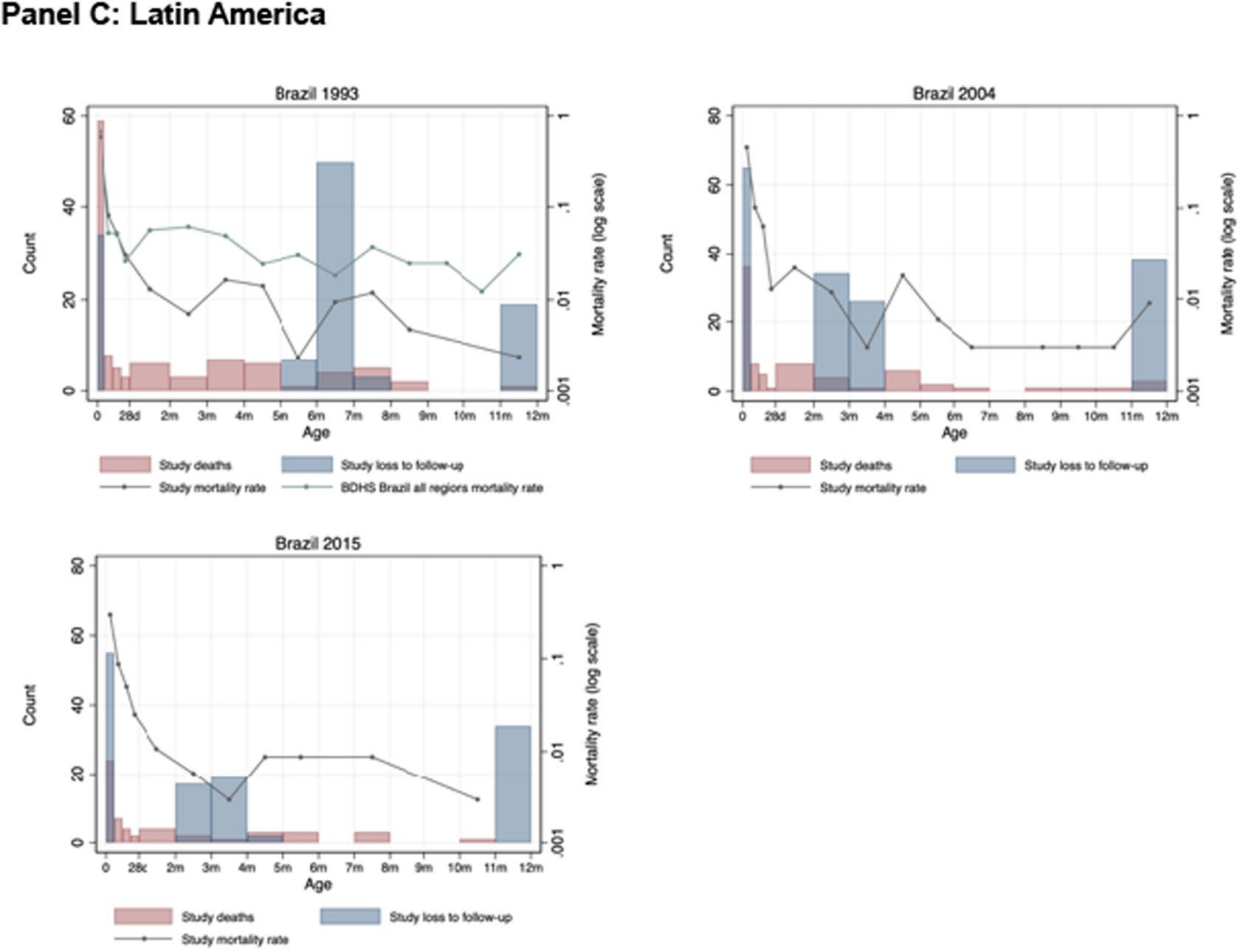
Distribution of age at death and loss to follow-up in neonatal or infant period by study. **A** Graphs include live births with complete vital registration data: India 2000: *n *= 14,147; Nepal 1999 *n *= 4130; Nepal 2011 *n *= 32,010; Philippines 1983: *n *= 3070 observations with complete data (*n *= 79 live births excluded for missing vital event data). All four of these studies were pregnancy cohorts. **B** Graphs include live births with complete vital registration data: Burkina 2004: *n *= 1321; Burkina 2006: *n *= 1102; Kenya 1992: *n *= 2332; Zimbabwe 1997: *n *= 14,108. Burkina Faso 2004 and 2006 and Kenya 1992 were pregnancy cohorts; Zimbabwe 1997 was a birth cohort. **C** Graphs include live births with complete vital registration data: Brazil 1993: *n *= 5248; Brazil 2004: *n *= 4219; Brazil 2015: *n *= 4270. Brazil 2015 was a pregnancy cohort; Brazil 1993 and 2004 were birth cohorts

LTF for newborns after delivery between day 0 and 27 ranged from 0.1% to 4.8%, while LTF between day 28 to one year ranged from 0.7% to 43.9% (Table 3, Fig. 1). In most studies, the reason for LTF was unspecified and, potentially due to out-migration. For three studies, reasons for LTF in the infant period were specified, including Nepal 1999 (LTF: *n *= 88, 98.9%; refusal: *n *= 1, 1.1%), Nepal 2011 (LTF: *n *= 1140, 94.8%; refusal: *n *= 62, 5.2%; maternal death: *n *= 1, 0.1%), and the Philippines 1983 (LTF: *n *= 246, 71.3%; refusal *n *= 44, 12.8%; multiple births (not followed according to study protocol): *n *= 55, 15.9%).

**Table 3:** Following and recording infant vital status and loss to follow-up in the neonatal and infant periods

Study	No. live births	No. neonatal deaths (0 to 27 days)	No. alive at 27 days	No. (%) LTF before 28 days	NMR (deaths per 1000 livebirths)	DHS NMR* (deaths per 1000 livebirths)	No. deaths among children 28 days–12 months	No. alive at 1 year	No. (%) LTF between 28 days and 12 months	IMR (deaths per 1000 livebirths)	DHS IMR* (deaths per 1000 livebirths)
*Asia*											
India 2000	12,936	449	12,461	26 (0.2%)	34.7	33	N/A	N/A	N/A	N/A	N/A
Nepal 1999	4130	174	3952	4 (0.1%)	42.1	43	80	3787	85 (2.2%)	61.5	64
Nepal 2011	32,121	1001	29,917	1203 (3.7%)	31.2	30	N/A	N/A	N/A	N/A	N/A
Philippines 1983	3149	38^1^	2960	151 (4.8%)	12.1	12	53	2713	194 (6.6%)	28.9	24
*Sub-Saharan Africa*											
Burkina Faso 2004	1337	24	1310	3 (0.2%)	18.0	29	63	1037	210 (16.0%)	65.1	67
Burkina Faso 2006	1225	25	1192	8 (0.7%)	20.4	29	36	633	523 (43.9%)	49.8	67
Kenya 1992 +	2332	54	2274	4 (0.2%)	23.2	24	293	1964	17 (0.7%)	148.8	130
Zimbabwe 1997	14,110	125	13,620	365 (2.6%)	8.9	16	779	10,958	1883 (13.8%)	64.1	29
*Latin America*											
Brazil 1993	5249	75	5140	34 (0.6%)	14.3	15	35	5026	79 (1.5%)	21.0	45
Brazil 2004	4231	50	4116	65 (1.5%)	11.8	15	28	3990	98 (2.4%)	18.4	24
Brazil 2015	4275	37	4183	55 (1.3%)	8.7	9	17	4094	72 (1.7%)	12.6	14

^1^Not included in the number of neonatal or infant deaths for Philippines 1983 are 10 deaths among women not enrolled because they delivered outside the study area, 16 deaths among enrolled out-migrants, 6 deaths among 27 sets of multiple births (1 died shortly after birth and 5 between months 2 and 9), and 6 deaths excluded because of missing date of death

^+^Not included in the number of live births for Kenya 1992 are 10 observations excluded due to missing data. Also excluded from analysis are 14 deaths with no known date of death

*NMR and IMR from closest DHS region and time period, specifically: India 2000 (DHS 2005/2006, Tamil Nadu Rural Region), Nepal 1999 (DHS 2006, Central Terai Region), Nepal 2011 (DHS 2016, Province 2 Rural Region), Philippines 1983 (DHS 1993, Central Visayas Region), Burkina Faso 2004 and 2006 (DHS 2010, Hauts Bassins Region), Kenya 1992 (DHS 2003, Nyanza Rural Region), Zimbabwe 1997 (DHS 2005, Harare Region), Brazil 1993 (DHS 1996, Brazil All Regions), Brazil 2004 (United Nations Inter-agency Group for Child Mortality Estimation (UN IGME) (2004.5), Brazil 2015 (United Nations Inter-agency Group for Child Mortality Estimation (UN IGME) (2015.5)

#### Date heaping

Heaping for the date of death was observed in Burkina Faso 2004 and 2006 (due to reliance on maternal recall), Zimbabwe 1997 (15th), Kenya 1992 (15th), and potentially also in Brazil 2015 and Nepal 1999 (1st and 15th) (Additional file 1: Appendix 2). There was no evidence of heaping for dates of the birth outcome in the 11 studies.

#### Shape of mortality curve

Figure 1 presents histogram distributions of the number of infants who died, the number of infants lost to follow-up, and log mortality rates for the first four weeks of life and months 2 to 12 for each study and the best matching DHS survey and region.

### External checks

#### Comparison with DHS

NMR among the cohort studies was relatively similar to the comparison group in Asia (DHS) and Brazil (national data from DHS for 1993 and United Nations Inter-agency Group for Child Mortality Estimation for 2004 and 2015). However, among the Africa studies, NMR was substantially lower in the study data compared to DHS, except for Kenya 1992, which was similar. In Asia, IMR was lower for Nepal 1999 and higher for the Philippines, relative to DHS. Among studies in Africa, IMR in Burkina Faso 2004 and 2006 was lower than DHS and much higher in Kenya 1992 and Zimbabwe 1997. In Brazil, IMR was lower than DHS with this difference decreasing from 1993, 2004, to 2015 (comparison was Brazil nationally).

## Discussion

Our analysis of 11 cohort studies identified field protocols that determine the appropriateness of vital event data for the purpose of mortality estimation. We found that missing birth and death outcomes—a source of bias if selection is associated with mortality risk—were influenced by several aspects of cohort study design and implementation. Several studies achieved low LTF in pregnancy and the neonatal period with no evidence of date heaping, likely due to frequent follow-up visits. Neonatal mortality rates between the external sources and the cohorts were similar in Asia and Latin America and substantially lower in most cohorts in Sub-Saharan Africa. Patterns of infant mortality varied by study and region between cohort studies and DHS comparison data. Potential reasons for these differences and their implications are discussed, while recognizing the absence of a single “gold standard” for mortality estimation.

Review of study design, population, and field protocols, as well as rates of LTF in pregnancy, suggest that studies enrolling pregnancies, rather than live births, are more likely to ascertain a high proportion of birth outcomes and less likely to miss very early neonatal deaths. The Nepal 1999 and the Burkina Faso studies achieved high follow-up of pregnancies with very few missing birth outcomes. Notably, Brazil 2015, a facility-based study, was able to attain a similar result. Nepal 2011 enrolled pregnancies or recorded birth outcomes for women not initially captured by pregnancy surveillance. This open cohort approach allowed for in-migration (and offset out-migration for the same reason) due to women returning to their maternal home for pregnancy and delivery, a common cultural practice in South Asia, especially among younger, nulliparous women. Nepal 2011’s high LTF in pregnancy and the neonatal period is also due in part to administrative censoring after study completion; a cause of missing data less likely to be associated with selection bias for mortality outcomes.

The specific protocols for pregnancy enrollment and follow-up influence whether a high proportion of birth outcomes are captured. Zimbabwe 1997, relied on a wide enrollment window (< 96 h of delivery) and enrolled women/infants only during daytime (potentially excluding women with obstetric complications); the substantially lower early neonatal mortality rate observed in this study is likely due in part to missed early deaths (mortality risk among HIV infected infants was lower in the early weeks of life suggesting missing deaths in this group) [27]. The Philippines 1983 conducted a follow-up survey after completing the primary study that found that many pregnancies, some that later resulted in an infant death, had been missed by pregnancy surveillance.

DHS FBHs are susceptible to missing and inaccurate vital event data for births and deaths, particularly at early ages, resulting from under-reporting of deaths and age misreporting [11]. Although thought to be less common due to use of prospective follow-up, there is also evidence that omissions of births and deaths are an important source of bias at early ages in HDSS sites, a result of time between surveys, recall bias, and the reliability of proxy respondents (i.e., person other than the mother) [28, 29]. Here the strengths of cohort studies—early and complete identification of pregnancies and frequent follow-up visits in pregnancy and the early neonatal period (e.g., as observed in Nepal 1999)—may offer a less biased source of data for estimation of fine strata mortality risks on the first days of life.

Generally, we found that cohort studies applied few inclusion/exclusion criteria; however, when utilized to address specific primary research questions, they can introduce selection bias into vital event data if associated with mortality risk. An example is Zimbabwe 1997, which excluded very low birth weight infants (< 1500 g), most likely leading to underestimation of early neonatal mortality.

Cohort studies had frequent field visits, especially those based in the community, often beginning with a census followed by prospective, house-to-house visits at varying intervals (e.g., Nepal 1999, 2011, and India 2000). In the Burkina Faso 2004 and 2006 studies, mothers and infants were seen every month at well-baby clinics at the health facility, leading to more missed follow-up visits, and longer maternal recall of date of death, than if visits had been conducted at the household. Or, in the case of facility-based studies, visits occurred daily to multiple health facilities or antenatal care centers (e.g., Brazil 2015). These approaches increase the likelihood that enrolled pregnancies will be followed to the birth outcome. Visits immediately and frequently after a birth outcome should be prioritized highly to avoid missed very early neonatal deaths, regardless of the study design; this is a major strength of cohort studies compared to DHS FBHs and HDSS.

Our data did not allow for the investigation of potential misclassification of stillbirths and neonatal deaths. This could be reliably done only in studies enrolling pregnancies. Evidence suggests misclassification of these outcomes can cause underestimation or overestimation of mortality rates, depending on the clinical and socio-cultural context. Further, if women can be followed early in pregnancy, then miscarriage rates will be more accurate. More accurate measures of gestational age, such as estimated by ultrasound examination, which is now more feasible and available in low-resource settings than in the past, rather than the less accurate dates of last menstrual period or postnatal assessment methods, will make estimation of stillbirth and miscarriage rates more accurate [30].

Cohort studies were largely unaffected by date heaping bias, except Burkina Faso 2004 and 2006, Kenya 1992, and Zimbabwe 1997. Date heaping in DHS FBHs is a cause of transferences of deaths from the early to late neonatal period due to heaping on day 7 of life [31]. Heaping has been associated with underestimation of neonatal and overestimation of postnatal mortality in HDSS [32], suggesting that cohort studies offer an advantage in their ability to reduce the impact of this bias on early mortality estimates. High-quality training and supervision of locally-resident data collectors utilized in these studies are reasons for this strength of cohort studies.

LTF varied across studies and the infant period but was generally low. Theoretically, LTF will only bias mortality rates if there is differential risk of death between those LTF and not LTF. Several cohort studies had < 1% LTF in the neonatal period (Nepal 1999, India 2000, Burkina Faso 2004 and 2006, Kenya 1992, and Brazil 1993), while others had around 3% or higher (e.g., Philippines 1983, Zimbabwe 1997, Nepal 2011), indicating increased potential for bias associated with more missed early deaths among LTF infants. Given small sample sizes of these studies, even a few missing deaths could significantly impact mortality rates.

Only rough comparisons between study and DHS mortality rates are possible given known biases with DHS and differences in geographical coverage areas. Cohort studies in Asia had similar NMR and IMR rates compared to DHS; this was observed even for the Philippines 1983, which experienced missing birth outcomes, LTF, and other biases. Data from the Philippines 1983, demonstrate a noticeable reduction in mortality risk at the first month of life followed by an increase at three months of life, peaking at six months. A follow-up survey identified 38 deaths among out-migrants, multiple births, and others that could not be included in the mortality analysis due to missing vital event data. Investigators reported finding misreported (later) dates of death (to avoid violation of government law related to late reporting of mortality outcomes), potentially leading to underestimated early, and overestimated late, mortality rates [33].

Studies in Africa, including Burkina Faso 2004 and 2006, had lower NMR than DHS, although there was better agreement in the postnatal period. One potential reason for this lower mortality rate, compared to DHS, could be the intense follow-up in the trial, including frequent visits study community health workers, high level of micronutrient intakes, and multiple antenatal care visits. Even in the absence of selection biases at enrollment, differences in the level of care delivered in the trial, compared to the general population, could affect representativeness of mortality estimates. The Kenya 1992 study had a relatively similar NMR to DHS over the study period. Zimbabwe 1997 study NMR mortality rates are much lower than the DHS rates, potentially a result of the study inclusion/exclusion criteria, and this relationship is inverted in the postnatal period. The increase in the mortality rate between two and five months in Kenya could result from waning passively transferred maternal antibodies against malaria, which contributed to a large burden of mortality and morbidity around this time [34, 35]. In Zimbabwe 1997, mortality rapidly increased from one to three months; reasons for this pattern could be missed early deaths; exclusion of LBW infants; or HIV infection, given 32% of enrolled women were HIV positive and this study took place before the availability of prevention of vertical transmission.

Our study had limitations. Studies included in this analysis were not identified through a systematic review, posing the possibility of selection bias associated with the design, protocols, and other methodological characteristics. Of note was the variation in study designs, field protocols, locations, and time periods across studies, which presented challenges for comparison between included studies and generalizability to other studies outside this analysis. These factors are likely not critical for internal validity in randomized trials or observational studies but can impact mortality patterns by age and sex. We did not evaluate the impact of trial interventions on mortality rates, nor could we evaluate the effects of seasonality and other external factors on mortality estimates or any effect of progressive intervention trials over many years in a single geographic site on mortality estimates.

We have described potential sources of bias in prospective cohort studies in Table 4. These include issues with pregnancy and birth outcomes and mortality estimation, such as missingness, loss to follow-up/out-migration, in-migration, misclassification, and date heaping and recall biases. The table also indicates the possible impact of these biases on mortality rates and proposes approaches to reduce these biases. The direction and magnitude of biases are often specific to the study design, site, and cultural context. Investigators should aim to understand local and cultural factors associated with potential biases and design customized strategies to reduce their impact. Investigators should be careful to note how the exclusion of certain participants could introduce selection bias (if associated with mortality risk) and how this differs from study of a special population, wherein mortality estimates may be unaffected by selection bias, but still non-representative of the underlying population. Quantitative validation studies comparing vital event data, FBHs, HDSS, and cohort studies, and the effects of various field protocols, should be the focus of future research to understand the potential for this underutilized resource for mortality estimation.

**Table 4:** Potential sources of bias in vital event data in population-based cohort studies

Source of bias	Potential bias on mortality estimates	Indicators to assess presence of bias	Approaches to reduce bias in cohort studies
*Pregnancy *and* birth outcomes*			
Missed pregnancies: Certain pregnant women, potentially eligible for a study, are missed by pregnancy surveillance	- Underestimate mortality Reason: Women missed by the survey may be more likely to experience stillbirth or neonatal/infant mortality	- Comparison of estimates to DHS, HDSS, or other data - Study protocols for identifying pregnancies through multiple avenues	- Conduct census or utilize vital registration and health care information systems - Enroll participants through multiple sources (e.g., ANC sites, health facilities, at home) - Conduct follow-up surveys
Birth under-reporting: Differential enrollment, loss to follow-up, or identification of the birth outcome for pregnant women who deliver at home vs. in a health facility	- Underestimate or overestimate mortality - Underestimate very early/early neonatal mortality Reason: Infants born at home may be less likely to be identified and included. They may be more likely to experience mortality due to reduced access to health care or lower socioeconomic status. In resource-limited settings with low facility delivery rates, women with a complicated pregnancy or delivery may be more likely to seek care, especially last minute, creating a spurious association between care seeking or facility delivery and adverse outcomes, such as stillbirth or early neonatal mortality [36]	- Facility delivery rate - Study protocols for identifying births at multiple sites and home - Comparison of characteristics of women delivering at home vs. facility	- Use community-based enrollment in settings without high facility delivery rate - Enroll participants through multiple sources - Ask participants about birth plan - Conduct early/frequent visits on day of birth if possible
Selection bias associated with loss to follow-up/out-migration, in-migration, or censoring: Certain pregnant women move from the home where they were originally enrolled and cannot be reached to obtain vital status and date of death if the infant died Conversely, certain pregnant women move into a study area and may be included in birth and mortality estimates Women still pregnant at the time of the study end date have an unknown birth outcome	- Underestimate mortality - Underestimate very early/early neonatal mortality Reason: Loss to follow-up due to out-migration in pregnancy is a common cause of selection bias. Women that are lost may differ in ways that impact mortality estimates. E.g., in some communities, women return to their parental homes late in pregnancy for delivery and the postnatal period. The characteristics of these women may differ from those who do not practice this custom in ways that affect birth and mortality outcomes Reason: Similarly, women who enter the study area due to in-migration may differ in ways that impact mortality estimates Reason: Some studies follow infants to a fixed age until the last infant has been followed, but others set an end date for the study. Deaths might occur among infants of censored women. However, this may not be associated with risk for mortality and therefore is not likely a significant source of bias	- Comparison of characteristics of women who were successfully followed vs. those who out-migrated or in-migrated - Comparison of characteristics of women censored vs. not censored	- Use comprehensive participant tracking (e.g., via digital technology or cell phone) - Conduct follow-up surveys for basic mortality information - Include in-migration to offset out-migration for women moving to parental homes to deliver in the study area - Carefully consider the potential biases associated with an open vs. closed cohort in the specific study context
Inclusion/exclusion criteria: Certain participants or special populations are excluded for the purpose of achieving the study’s primary aim	- Underestimate or overestimate mortality - Generate unbiased estimates of mortality for a special, non-representative population Reason: Exclusion of certain participants could introduce selection bias if their mortality risk differs from those who are included. This includes exclusion of certain participants not reached within some time frame after delivery (e.g., < 72 h). If a certain special population is the focus of a study, mortality estimates may be unaffected by selection bias, but still non-representative of the underlying population	- Review evidence on mortality risk in included vs. excluded populations - Compare characteristics in those included vs. excluded	- Limit exclusion criteria if possible - Collect key characteristics and vital event data on all participates, to allow for comparisons, even if a subset of participants are excluded from the primary study and/or analysis - Assess and report the potential generalizability of mortality estimates from special populations and how they may differ from the underlying population
*Mortality outcomes*			
Misclassifying very early neonatal deaths: Differential misclassification of neonatal deaths as stillbirths or vice versa	- Underestimate or overestimate very early neonatal mortality Reason: Very early deaths occurring at home may be less likely to be correctly classified and reported (may be misclassified as stillbirths or vice versa). There are many reason this happens, including absence of skilled birth attendance at delivery and stigma or other reluctance related to reporting neonatal deaths	- Compare estimates and ratio of stillbirths to neonatal deaths to DHS, HDSS, or other data	- Conduct early/frequent visits on day of birth if possible - Use verbal autopsy surveys to classify antepartum stillbirths, intrapartum stillbirths, and early neonatal deaths
Under-reporting of neonatal or infant deaths: Neonatal or infant deaths are not reported or hidden from data collectors and/or health local authorities	- Underestimate mortality - Underestimate very early neonatal mortality Reason: In many settings there is stigma associated with reporting neonatal or infant deaths that could result in substantial under-reporting of birth outcomes. Under-reporting may also be associated with specific participant characteristics, particularly those indicative of disadvantaged populations	- Examine mortality pattern and rates (e.g., across first day, week, month, and year of life) - Compare estimates to DHS, HDSS, or other data	- Conduct comprehensive follow-up and participant tracking to reduce number of missed mortality outcomes - Describe cultural factors associated with potential bias and design customized strategies to reduce their impact
Selection bias associated with loss to follow-up/out-migration, in-migration, or censoring: Certain mothers and infants move from the home where they were originally enrolled and cannot be reached to obtain vital status and date of death if the infant died Conversely, certain mothers and infants move into a study area and may be included in mortality estimates Infants not having reached 28 days or 1 year of life (or other benchmark) at the time of the study end date have an unknown vital status at that time point	- Underestimate mortality - Underestimate very early neonatal mortality Reason: Loss to follow-up due to out-migration is often the most common cause of selection bias in infancy. Mothers and infants that are lost may differ in ways that impact mortality estimates. Live birth cohorts that allow enrollment of newborns beyond the day of delivery may introduce substantial bias, leading to underestimated very early and early neonatal mortality Reason: Similarly, mothers and infants who enter the study area due to in-migration may differ in ways that impact mortality estimates Reason: Some studies follow infants to a fixed age until the last infant has been followed but others set an end date for the study. Deaths might occur among censored infants. However, this may not be associated with risk for mortality and therefore is not likely a significant source of bias	- Compare maternal or infant characteristics of participants successfully followed vs. those who out-migrated - Compare numbers of deaths and LTF by age category - Comparison of characteristics of infants censored vs. not censored	- Use comprehensive participant tracking (e.g., via cell phone) - Conduct follow-up surveys for basic mortality information - Exclude mothers and infants who are censored from mortality estimates - Utilize survival analysis to include and appropriately apportion time contributed by censored infants - Carefully consider the risks of missed birth outcomes and out-migration, and, especially, in-migration in the neonatal period, among live birth cohorts, which do not have the benefits of pregnancy enrollment
Date heaping and other recall error: Dates of death reported by mother, parents, or data collectors are sometimes rounded up to the 15th or 30th of the month when being recorded during data collection due to recall bias	- Underestimate of early or infant mortality Reason: Rounding up of dates of death to 15th or 30th of the month could shift deaths above age specific cut-offs for mortality estimates, such as 28 days or 1 year, reducing rates for early mortality categories	- Create histographs to explore presence of date heaping	- Use locally appropriate methods to improve date recall (e.g., event calendars) - Increase frequency of follow-up visits - Consider other methods to improve recall (e.g., diary for important dates) - Apply analytical techniques to adjust date heaping

## Conclusion

Prospective, population-based cohort studies that followed certain protocols can yield high-quality vital event data to contribute meaningfully to our understanding of mortality patterns of infants in LMIC settings (Table 5). These included enrolling pregnancies, limiting exclusion criteria potentially associated with mortality, capturing a high proportion of birth outcomes, immediate and frequent follow-up after delivery, and identifying and reducing other biases (e.g., related to the stigma of reporting a death) and data error issues (e.g., heaping). Cohort studies offer strengths not found in DHS FBHs or HDSSs, particularly immediate and frequent follow-up after the pregnancy outcome. Our results suggest that population-based cohort studies could provide high-quality vital event data for mortality estimation and understanding detailed patterns of mortality by age, particularly early in the neonatal period.

**Table 5:** Recommended protocols for collection of high-quality vital events data for mortality estimation in population-based birth cohort studies

1	Identify a high proportion or representative sample of pregnancies (or live births) in a geographic area to avoid selection bias associated with place of delivery
2	Enroll pregnant women, rather than live births at the time of delivery, to capture more live births and early deaths and reduce under-reporting of stillbirths and misclassification of neonatal deaths
3	Consider the impact of an open or closed cohort on mortality estimates given patterns of out-migration and in-migration behaviors in the study population
4	Minimize inclusion/exclusion criteria restrictions (e.g., exclusion of multiple births) on the study population for which vital event data is collected to avoid selection bias and reduce impact on generalizability associated with special populations
5	Attempt to capture vital information on pregnancy outcomes as quickly as possible (i.e., on the day of birth) after the occurrence of the birth outcome to avoid missing deaths (even if immediate follow-up is not required for the study’s primary aim)
6	Understand local reasons for misclassification of stillbirths and neonatal deaths and utilize staff training and study protocols to reduce this bias
7	Train study staff to avoid common epidemiologic biases and data collection errors that affect mortality estimates, such as reporting biases (e.g., recall bias or bias due to stigma of reporting a death) or date heaping
8	Reduce missing birth outcomes and infant vital status data by closely tracking participants through frequent visits, using digital technologies if possible, to reduce selection bias associated with loss to follow-up
9	Utilize post hoc analytical techniques to explore for and report on selection and reporting biases, such as date heaping graphs or comparison of participants fully followed vs. participants lost to follow-up

## Supplementary Information

**Additional file 1.** Supplementary Tables and Figures.

## Data Availability

Not applicable.
