# Supplementary material for: Quality of vital event data for infant mortality estimation in prospective, population-based studies: an analysis of secondary data from Asia, Africa, and Latin America
Source: Popul Health Metr. 2023 Jul 28;21:10. doi: 10.1186/s12963-023-00309-7 (PMC10375772; doi:10.1186/s12963-023-00309-7)
Supplement: Supplementary file 1 — Additional file 1. Supplementary Tables and Figures. [file 12963_2023_309_MOESM1_ESM.docx]

**Supplementary material**

**Appendix 1: Number of pregnancies with unknown birth outcome by reason for missing information for the seven birth cohort studies that enrolled pregnancies**

| **Study** | **Refusal** | **Loss to follow-up^*^** | **Maternal death** | **Data issue** | **Total** |
| --- | --- | --- | --- | --- | --- |
| Nepal 2011 | 309 (0.7%) | 5,560 (13.1%)~ | 7 (<0.1%) | 1 (<0.1%) | 5,877 |
| Nepal 1999 | 1 (<0.1%) | 2 (<0.1%) | 4 (0.1%) | 0 (0.0%) | 7 |
| Philippines 1983 | 94 (2.5%) | 372 (10.0%) | 0 (0.0%) | 16 (0.4%) | 482 |
| India 2000 | 0 (0.0%) | 765 (5.5%) | 6 (0.0%) | 0 (0.0%) | 771 |
| Burkina Faso 2004 | 0 (0.0%) | 40 (2.8%) | 3 (0.2%) | 2 (0.1%) | 45 |
| Burkina Faso 2006 | 0 (0.0%) | 23 (1.8%) | 3 (0.2%) | 1 (0.1%) | 27 |
| Brazil 2015 | 51 (1.2%) | 7 (0.2%) | 0 (0.0%) | 46 (1.1%) | 104 |

***** Loss to follow-up includes outmigration, participant unreachable, or participant missed by birth outcome surveillance.

~ In Nepal 2011, 3,101 (55.8%) of pregnancies lost to follow-up were censored due to the end of the study.

**Appendix 1:** Numbers are the total participants with an unknown birth outcome by reason for missing information and study. Percentages indicate the number of unknown outcomes for a specific reason as a proportion of the total identified pregnancies for each study.

**Appendix 2: Date heaping graphs for dates of death and dates of birth outcome**

***Frequency of dates of death for mortality in the infant period***

**Panel A: Asia**
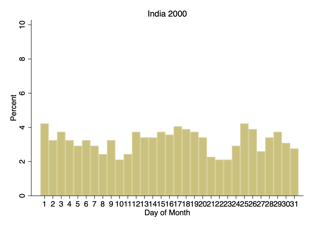


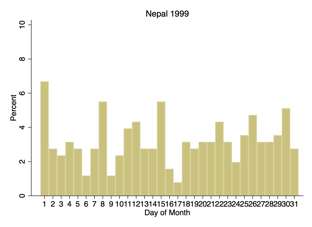


**
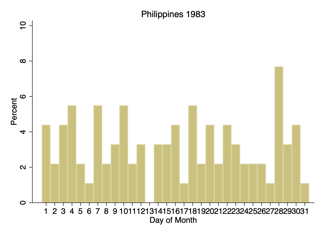
**

**
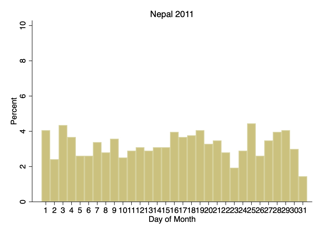
**

**Panel B: Sub-Saharan Africa***

**
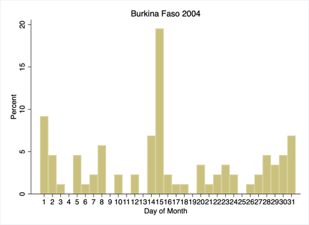
** **
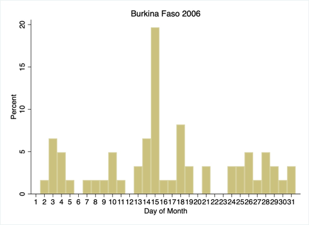
**


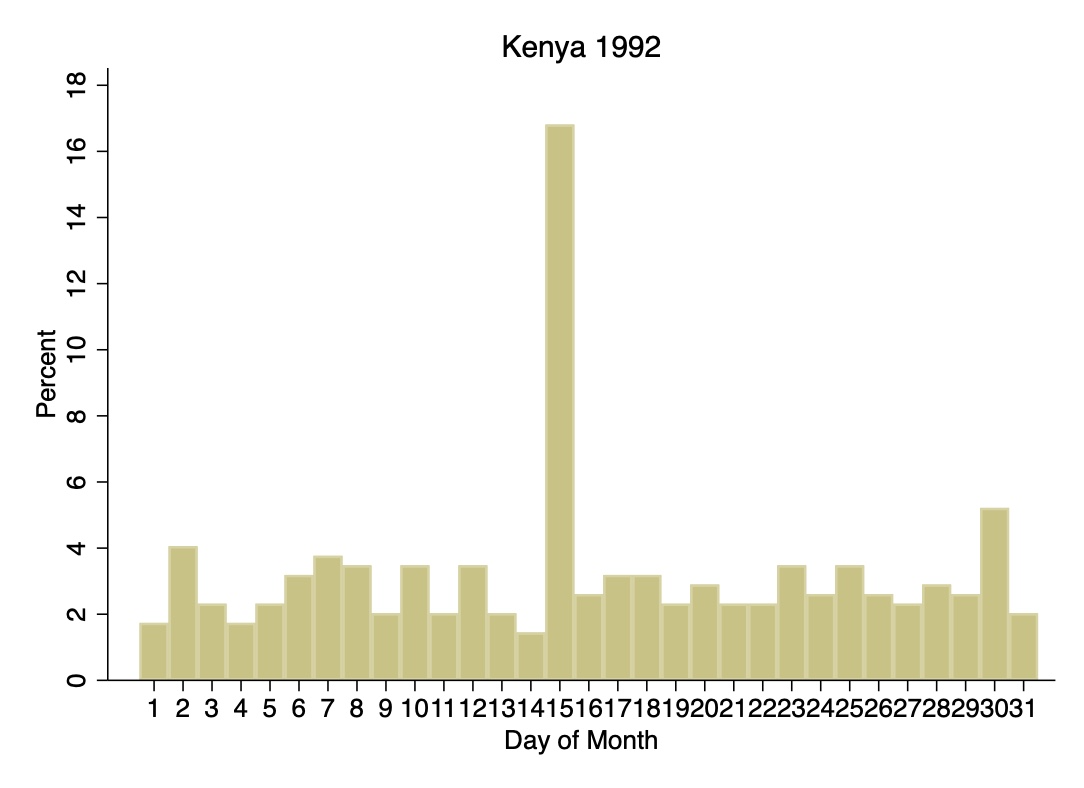
**
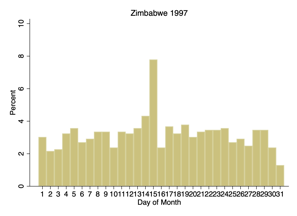
**

* Note: Burkina Faso 2004 and 2006 and Kenya 1992 Y axis scale ranges from 0 to 20 percent.

**Panel C: Latin America**

**
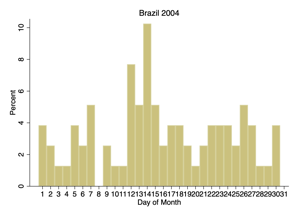
**
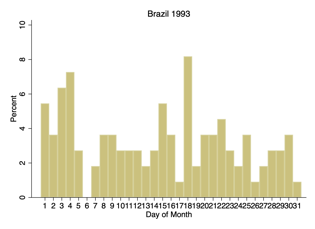


**
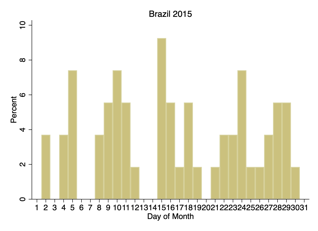
**

***Frequency of dates of birth outcome***

**Panel A: Asia**


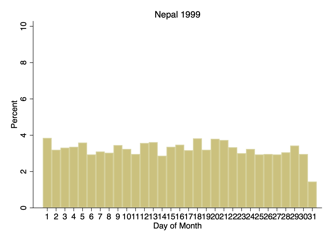

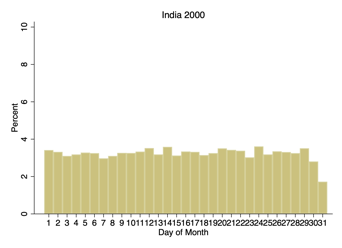


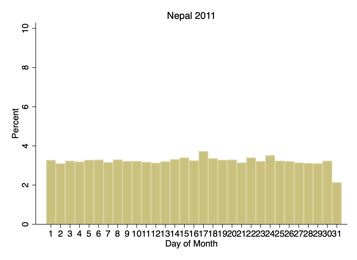


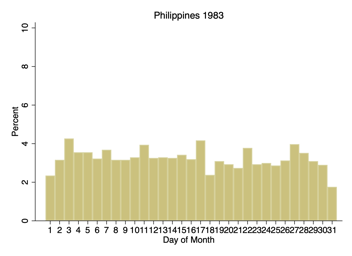


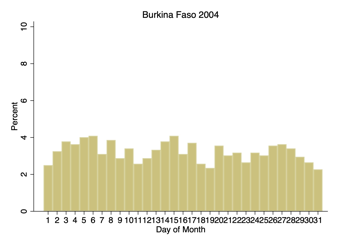
**Panel B: Sub-Saharan Africa**


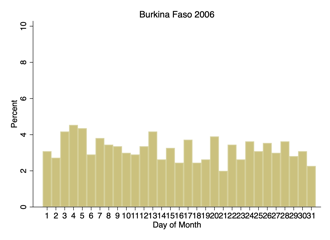


**
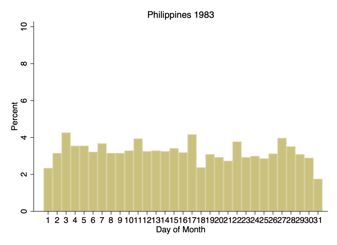
**

**
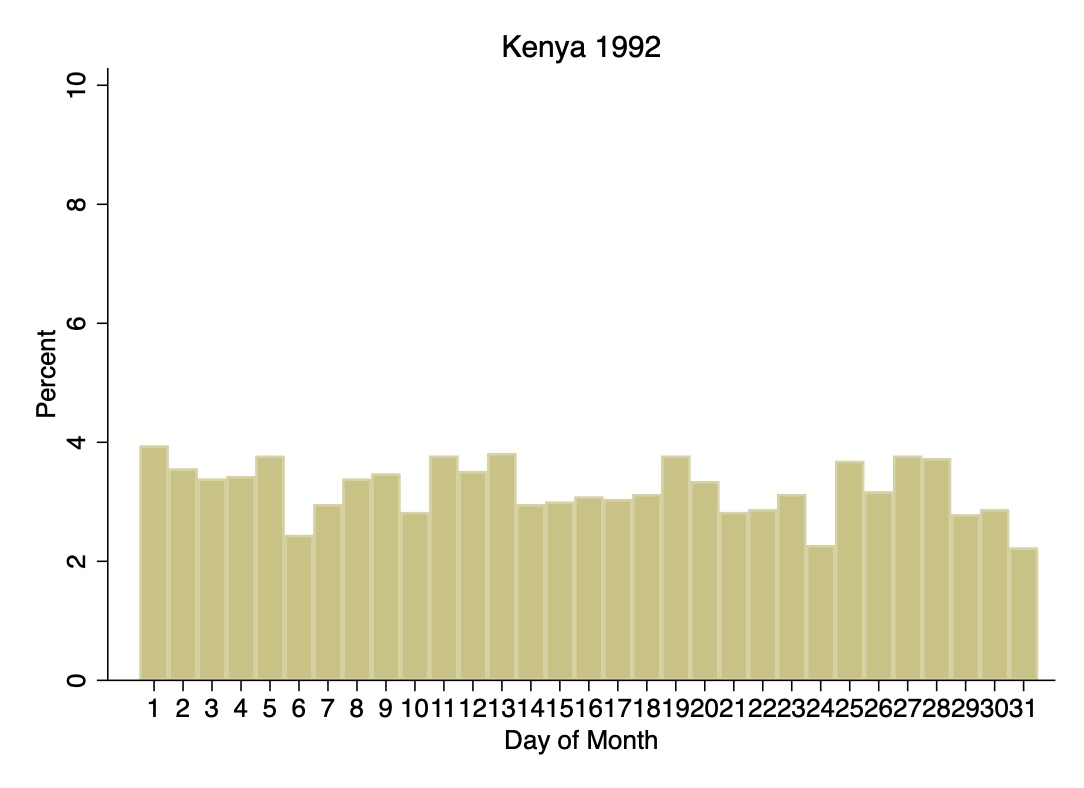
**

**Panel C: Latin America**

**
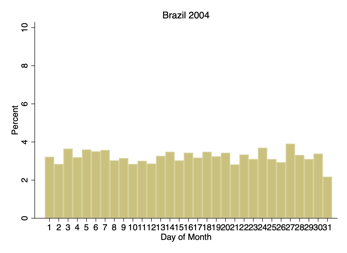
**
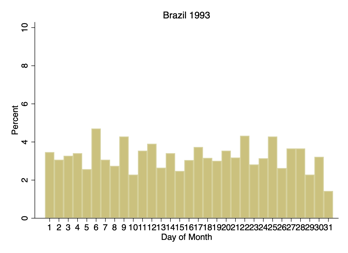


**
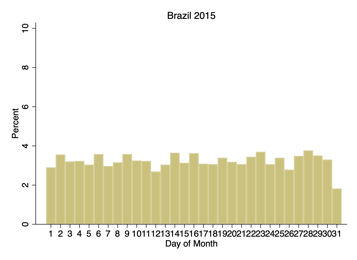
**

**Appendix 2:** Date heaping histograms display the distribution of days of the month for dates of death and birth, respectively, for each study.

**Appendix 3: Example flowchart for collecting high-quality estimates of child mortality**

**
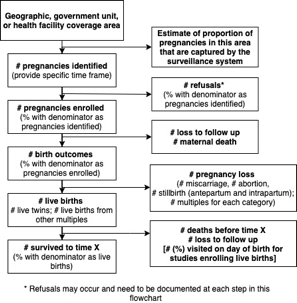
**

**Appendix 3:** This flowchart represents a generic structure to document essential protocols of population-based pregnancy and birth cohort studies for the purpose of identifying and recording pregnancies, birth outcomes, and mortality outcomes. The aim of this documentation should be to clearly indicate the number of, and time point when, pregnancies, birth outcomes, and infants, particularly in the first 24 hours of life, might be lost to follow-up, resulting in missed deaths and, consequently, inaccurate (lower) mortality estimates.
